# Hybrid Nanoporous Gold–Cyclodextrin Nanosponge Platforms for Enhanced Model Contaminant Detection

**DOI:** 10.1021/acsapm.6c00986

**Published:** 2026-05-11

**Authors:** Adrián Matencio, Federico Scaglione, Miriam Birolo, Alberto Rubin-Pedrazzo, Paola Rizzi, Francesco Trotta, Fabrizio Caldera

**Affiliations:** † Department of Chemistry and NIS - INSTM, 9314 University of Turin, Via P. Giuria 7, Turin 10125, Italy; ‡ Departamento de Bioquímica y Biología Molecular-A, Facultad de Biología, Universidad de Murcia−Regional Campus of International Excellence“Campus Mare Nostrum”, Murcia E-30100, Spain

**Keywords:** cyclodextrin-based nanosponges, nanoporous gold, lipoic acid, pollutant, sensor

## Abstract

The development of hybrid materials combining functional polymers with nanostructured metals has gained significant attention for environmental and sensing applications. In this study, we present a proof-of-concept demonstration of the first-ever binding of a nanosponge polymer (NS) to nanoporous gold (NPG). The polymer was functionalized with a biodegradable lipoic acid linker, allowing efficient attachment to gold surfaces via sulfide bonds. To achieve successful binding, reducing agents were required during NS synthesis to generate free thiol (−SH) groups. Two synthesis approaches were tested, and optimal binding was confirmed with a polymer-to-surface ratio of 1.1 × 10^–3^ mg/mm^2^, as validated by surface-enhanced Raman spectroscopy (SERS) and colorimetric analysis. Additionally, the functionalized NPG was tested for its ability to detect methylene blue, a model contaminant, showing a 19% higher response compared to unmodified gold. These findings establish a foundation for further development of hybrid materials aimed at the detection and potential removal of emerging pollutants from water.

## Graphical abstract

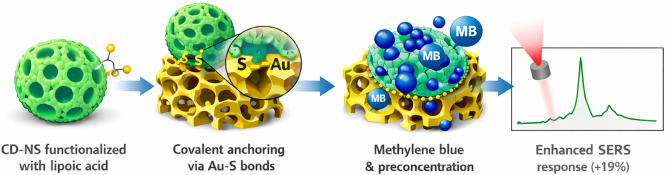

## 1 Introduction

Water, an essential resource for all living organisms, is increasingly threatened by contamination arising from human activity, climate change, and demographic growth. In particular, the presence of emerging pollutants (EPs), chemical compounds that are often unregulated and insufficiently understood in terms of their environmental and health impact, poses a major challenge for water quality management. These substances, including pharmaceuticals, pesticides, and personal care products, are frequently detected at low concentrations yet persist in aquatic environments and may bioaccumulate over time. As a result, there is a growing need not only for efficient removal strategies but also for advanced sensing platforms capable of detecting and monitoring such contaminants in complex aqueous systems. In this context, hybrid materials that combine selective recognition elements with responsive or transducing components have attracted increasing attention as promising solutions for pollutant monitoring and environmental sensing applications.^1^

Traditional wastewater treatment plants are not designed to address complex chemical pollutants, such as EPs. While primary and secondary treatments can remove some contaminants through biological degradation or adsorption, many EPs survive even tertiary treatments. Existing chemical methods, such as precipitation, adsorption, and oxidation, can be enhanced by using modern materials, including biopolymers and nanomaterials, for water remediation.^2,3^ Cyclodextrin nanosponges (CD-NSs), with their environmental compatibility, high complexation ability, and structural adaptability, have shown significant promise in capturing pollutants like aromatic hydrocarbons and heavy metals.^4–7^

This article builds on these advancements, focusing on an innovative detection system for water pollutants. It integrates nanoporous gold (NPG) and CD-NS, connected via lipoic acid as a molecular “bridge.” Cyclodextrin nanosponges, derived from cross-linked cyclodextrins, combine the host–guest chemistry of CDs with enhanced stability and adsorption capacity. The NS functionalization with lipoic acid adds a sulfur-based group to interact with the NPG surface, enabling dual functionality: pollutant sequestration and monitoring via the metal’s optical properties.^8^

Nanoporous gold, with its stable three-dimensional scaffold and high surface area, provides a robust platform for surface-enhanced Raman spectroscopy (SERS). This technique allows highly sensitive detection of pollutants, leveraging the unique morphology and plasmonic properties of NPG to amplify molecular signals.^9–11^ Together, the CD-NS and NPG systems offer an eco-friendly, high-performance solution for pollutant detection and environmental remediation.^10,12^

In this context, the present work is conceived as a proof-of-concept study aimed at demonstrating the feasibility of integrating CD-NSs with NPG and evaluating their combined behavior using a model compound. Through this approach, the study provides a basis for the further development of more advanced systems for tackling emerging water pollutants.

Considering all the previously said, several points were studied:

Synthesis and characterization of a CD-NS functionalized with lipoic acid.
Bond between CD-NS-Lipoic and NPG (the sensor).
Complexation of a model pollutant (methylene blue) with CD-NS-lipoic.
Evaluation of the detection of methylene blue (MB) concentration with the new sensor.

## 2 Experimental Section

### 2.1 Materials

The β-cyclodextrin (β-CD, CID 444041) was kindly provided by Roquette Freres (Lestrem—France). NPG was synthesized from the dealloying of an amorphous precursor, as detailed below. Lipoic acid (LA, CID 6112), 1,1′-carbonyldiimidazole (CDI, CID 68263), methylene blue (MB, CID 6099), dithiothreitol (DTT, CID 19001), and the remaining reagents were purchased from Merck/Sigma-Aldrich (Milan, Italy).

### 2.2 Equipment and Experimental Procedure

#### 2.2.1 Preparation of the Nanoporous Gold

The synthesis procedure for NPG has been optimized in earlier studies; a concise overview is presented here.^10,13^ A master alloy ingot with the nominal composition Au_20_Cu_48_Ag_7_Pd_5_Si_20_ was prepared by arc melting high-purity elements (99.95%–99.99%) in a Ti-gettered argon atmosphere using a Buehler electric arc furnace (Edmund Bühler GmbH, Bodelshausen, Germany). This composition was selected for its excellent glass-forming ability, yielding a suitable amorphous precursor.^14^ Rapid solidification of the ingot was carried out using the melt-spinning technique (Edmund Bühler GmbH, Bodelshausen, Germany).^15^ The molten alloy was ejected from a quartz crucible through a 2 mm nozzle onto a copper wheel rotating at 25 m/s in an argon atmosphere, producing amorphous ribbons ∼25 μm thick and 2 mm wide.

The as-quenched Au-based amorphous ribbons were chemically dealloyed in a water bath containing 10 M HNO_3_ and 0.5 M HF at 70 °C for 5 h. After dealloying, the samples were thoroughly rinsed with double-distilled water, air-dried, and stored in clean vials until further characterization. Prior to functionalization, NPG strips, approximately 3 cm in length, were thoroughly cleaned using a Piranha solution (7:3 v/v mixture of sulfuric acid and hydrogen peroxide), followed by extensive rinsing with ultrapure water until a neutral pH was achieved.

The surface morphology of NPG was examined via Scanning Electron Microscopy (SEM) (TESCAN, Brno, Czech Republic), operated at an accelerating voltage of 20 keV, coupled with Energy Dispersive X-ray Spectroscopy (EDS) (Oxford Ultim-Max 100, Oxford Instruments, Abingdon, UK) to assess elemental composition. The average ligament size of the NPG was estimated by measuring 200 ligaments at their narrowest necks using the open-source ImageJ software.^16^ The structural characterization of both the as-quenched amorphous ribbon and NPG was conducted using a Panalytical X’Pert X-ray diffractometer (Panalytical, Almelo, The Netherlands) in Bragg–Brentano geometry, employing Cu Kα radiation. Diffraction peaks were indexed using the X’Pert HighScore software (version 2.2c (2.2.3)).

#### 2.2.2 Synthesis of β-CD Nanosponges (βNS-CDI 1:8)

The cross-linked β-cyclodextrin (βCD) nanosponges were prepared according to procedures previously reported in the literature,^17,18^ with slight modifications. The synthesis was carried out using a planetary ball mill (PM200 High-Speed Planetary Ball Mill, Retsch) through a one-step, solvent-free reaction. An anhydrous β-cyclodextrin to 1,1′-carbonyldiimidazole (CDI) molar ratio of 1:8 was maintained throughout the process. Specifically, 3.75 g of β-cyclodextrin was placed inside a 50 mL milling jar containing ten zirconia balls with a diameter of 1 cm, and the appropriate amount of CDI was added to each batch to achieve the desired molar ratio. Additionally, the functionalization was carried out by directly adding to the synthesis a 0.1 molar ratio (with respect to cyclodextrin) of lipoic acid (LA, NS-LA 1:8:0.1), either alone or in combination with the same molar ratio of dithiothreitol (DTT, called RNS-LA 1:8:0.1:0.1, where R means “reduced”).

After 3 h of sun wheel rotation at 600 rpm, changing the direction from clockwise to anticlockwise every 15 min, the reaction was completed. The measured external temperature of the jars was around 60 °C. After the synthesis a finely ground white/yellowish powder was obtained; subsequently, the powder was dispersed in water and washed several times with deionized water and acetone. All synthesized nanosponges were extracted with Pressurized Solvent Extraction (PSE, SpeedExtractor E-914 from Buchi) with acetone, to remove the residual imidazole from the NS structure and unreacted material. Figure 1 schematically represents the synthesis pathway for the preparation of β-CD nanosponges.

**Figure 1:**
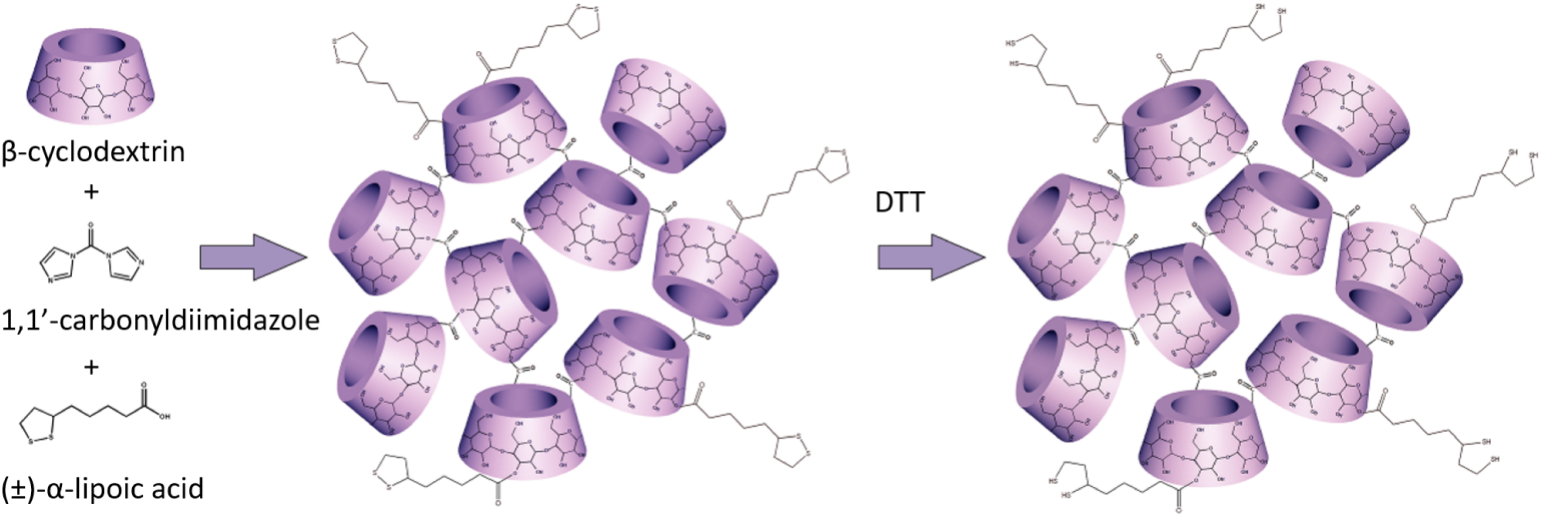
Schematic representation of the stepwise procedure for the synthesis of RNS-LA.

#### 2.2.3 Cyclodextrin-Based Nanosponge Quantification

The phenolphthalein colorimetric method for β-CD was adapted.^19^ A phenolphthalein solution was prepared by adding 2 mL of a 3 mM solution in EtOH to 98 mL of NaHCO_3_ buffer 125 mM at pH 10.5. 1 mL of this solution was added to 0.1 mL of NS suspension at different concentrations (0–1500 ppm), and 0.1 mL of Tris-Buffer pH 8, 0.1 mM. The sample was mixed for 1 h, centrifuged at 2500 rpm for 5 min and the supernatant was measured at 550 nm.

#### 2.2.4 Nanoporous Gold Bonding

Segment preparation: Different segments of 60 mm^2^ were washed using a 0.01 M Tris-HCl buffer solution at pH 8 for 30 min to remove excess acid under gentle agitation. They were then washed for 10 min with 2 cycles of Milli-Q water.

Binding: A suspension of RNS-LA was left in the presence of the segment for 24 h at 55 °C under gentle agitation. The temperature was kept constant using a sand bath. The completion of the reaction was monitored using the method described in Section 2.2.3.

#### 2.2.5 Sensor Proof of Concept, Methylene Blue Complexes with RNS-LA_NPG

Direct complexation: To understand the possible interaction between MB and RNS-LA, the inclusion complex was prepared. Briefly, a 1:10 weight ratio of MB:RNS-LA was mixed in Milli-Q water for 24 h in darkness. The complex was purified by filtration, dried, and stored in darkness. To determine the amount of sample produced, a desorption study in pure EtOH was performed. Samples of known weight were left in the presence of EtOH for 10 min until the color disappeared. The concentration was analyzed using an appropriate dilution and a calibration line (*R* ^2^ > 0.99).

The Removal Efficiency and Adsorption capacity were calculated according to the following equations:

1 $$RE\;\left(\%\right)=\frac{Complexed\;MB}{Total\;MB}\times 100$$

2 $$AC\;\left(\%\right)=\frac{Complexed\;MB}{mass\;of\;MBd}\times 100$$

Sensor simulation: The composites were incubated with a solution of MB in 0.8% NaCl and gently agitated. The supernatant was analyzed at 665 nm after 0.5 and 24 h, and the concentration was determined using a calibration curve of MB (*R* ^2^ > 0.99). The concentration inside was calculated by subtracting the amount remaining in the solution after that time from the initial quantity.

#### 2.2.6 Material Characterization: TGA, FTIR, Elemental Analysis, Particle Size, and SERS

TGA: TGA analysis was carried out on a TA Instruments Hi-res Q500 Thermogravimetric Analyzer (New Castle, DE, USA). TG Analysis parameters: nitrogen flow, heating rate 10 °C/min, RT to 700 °C in an air atmosphere.

FTIR: A PerkinElmer Spectrum 100 FT-IR spectrometer (Waltham, MA, USA) with an ATR sampling accessory was used for FTIR-ATR (attenuated total reflection) characterization. IR spectra on dried powders were recorded on a PerkinElmer Spectrum 100 FT-IR spectrometer with 16 scans.

Particle size: The particle size and polydispersity index were studied by Dynamic Light Scattering (DLS) using a Malvern Zetasizer Nano instrument at a fixed scattering angle of 90°. All the samples were suitably diluted (from 1000 to 1 ppm) by Milli-Q water and analyzed at 25 °C.

Elemental analysis: RNS-LA 1:8:0.1:0.1 was analyzed by the CHNS-O Analyzer (FLASHEA 1112 series).

SERS: measurements were conducted with a Renishaw inVia Raman Microscope (Renishaw, Wotton-under-Edge, England) using a 785 nm laser line, selected to match the plasmonic response of the NPG. A 50× ULWD objective was employed, resulting in an estimated laser spot size on the order of a few micrometers (≈2 μm). The laser power at the sample surface was kept low (0.05% of the nominal laser power ≈0.1 mW) to minimize local heating effects. Spectra were acquired with an integration time of 20 s and accumulations of 5 spectra. The reproducibility of the sensor was confirmed through duplicate mapping acquisition each conducted with a newly synthesized sample. The spectrometer was calibrated before measurements using the Raman band of a silicon wafer at 520 cm^–1^. It should be noted that no SERS enhancement factor (EF) or quantitative performance metrics were determined in this study, as no direct comparison with non-SERS Raman measurements under identical conditions was performed. Therefore, the SERS analysis is limited to qualitative observations and is intended to assess the feasibility of the proposed hybrid system.

#### 2.2.7 Statistical Analysis

The experiments were repeated at least three times, with the exception of TGA and FTIR, which were performed only one time. The statistical analysis was done using Social Science statistics (https://www.socscistatistics.com/) by using for all the samples the mean standard deviation (SD). The degree of significance was calculated using the *p*-value. Statistical significance was determined by *p* values of <0.05.

## 3 Results and Discussion

### 3.1 Characterization of NPG

The NPG sample, synthesized as previously described, was subsequently characterized by SEM and EDS analyses. Figure 2a shows the morphology of the NPG, consisting of an interconnected network of pores and ligaments. The inset displays a close-up view of the ligaments, which are composed of multiple knobby and rough grains, a typical feature of NPGs derived from amorphous precursors. The size of the multigrain ligaments, measured at the narrowest neck, has an average ligament size of 65 ± 8 nm.

**Figure 2:**
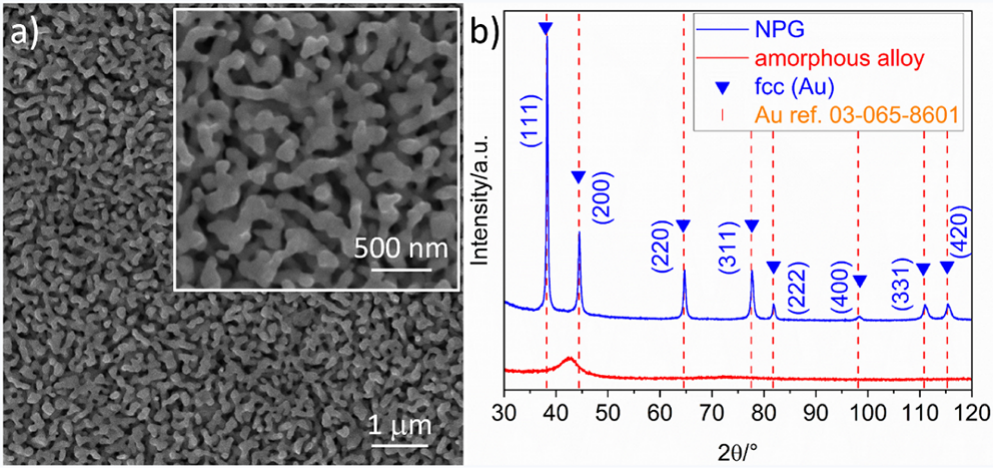
a) SEM image of NPG; in the inset, a close-up view of the ligament’s morphology. b) XRD pattern of NPG (blue line) and of the amorphous precursor ribbon (red line).

Moreover, EDS analysis reported in Table S1 in the Supporting Information indicates that Au is the dominant element, with only trace amounts of other elements detected: Cu (5.3 at. %), Ag (0.1 at. %), Pd (0.3 at. %), and Si (1.0 at. %). These residual elements are likely embedded in a solid solution within the Au ligaments as a consequence of the dealloying process.

The structural characterization of both the amorphous precursor and NPG is presented in Figure 2b. The red-patterned curve displays a single broad halo, typical of the amorphous precursor. In contrast, the blue-patterned curve for NPG shows the transformation of the amorphous phase into a crystalline structure during dealloying, evidenced by the emergence of distinct face-centered-cubic (fcc) peaks, corresponding to the Au-rich solid solution.

### 3.2 Synthesis and Characterization of βNS-CDI 1:8, NS-LA 1:8:0.1, and RNS-LA 1:8:0.1:0.1

On the basis of previously reported protocols,^17,18^ the feasibility of introducing LA was studied. Two different polymers were prepared, a polymer introducing LA directly in the synthesis (NS-LA 1:8:0.1) and a second one where the reducing agent DTT was also introduced (RNS-LA 1:8:0.1:0.1), to enable the availability of all the -SH.

The polymers were characterized by FTIR and TGA (Figure 3 a and b). The FTIR analysis reported in all the cases a band around 1600–1800 cm^–1^ due to the vibration of the ester bond, as occurs.^4,20^ Minimal differences were found in the area of 1000 cm^–1^ where the peak at 1024 increased on intensity, possibly due to the additional existence of C–O bonds by the presence of LA.

**Figure 3:**
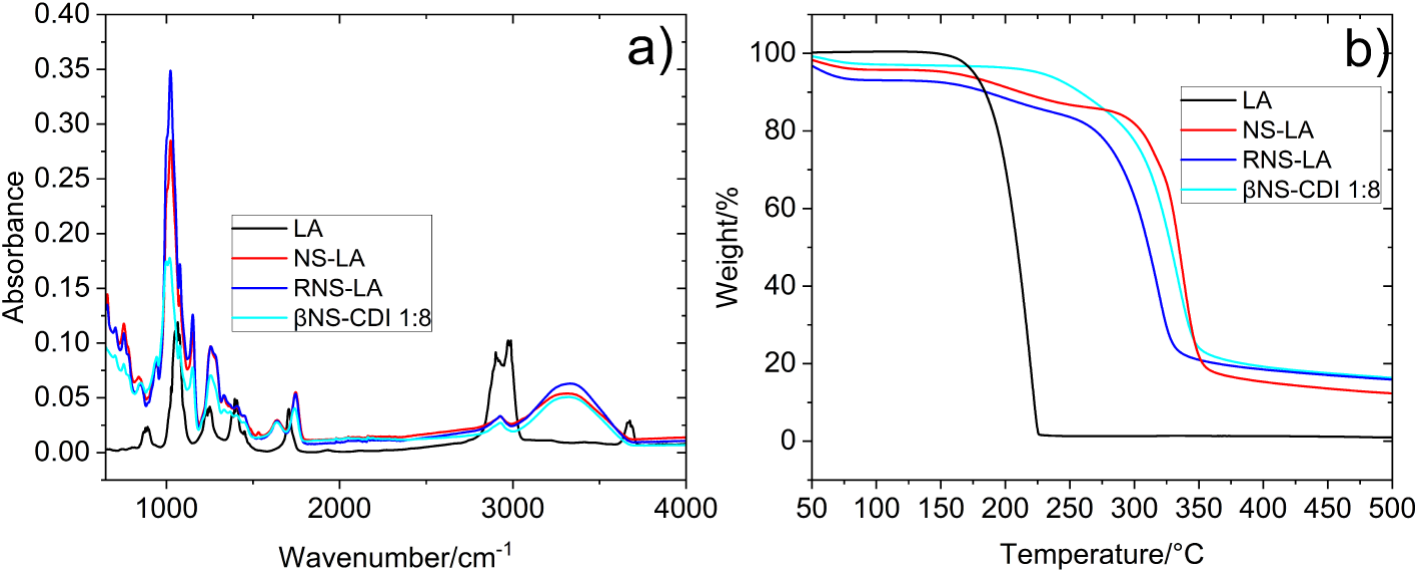
a) FTIR spectra of LA, NS-LA, and RNS-LA. b) TGA thermogram of LA, βNS-CDI 1:8, NS-LA, and RNS-LA.

TGA results of βNS-CDI 1:8 (Figure 3b, black line) present the classical profile of the base polymer, βNS-CDI 1:8, with a slight decrease before 100 °C due to hydration water, and a dTGA maximum around 333 °C due to the polymer degradation.^4,20^ However, both NS-LA (red line) 1:8:0.1 and RNS-LA (blue line) 1:8:0.1:0.1 showed different degradation compared to the base material (first RNS-LA at 318 °C, second NS-LA at 337 °C). The introduction of LA in the structure can modify the strength of the network affecting its thermal stability. On the other hand, the intramolecular R–S–S–R′ bonding between two different units of LA in NS-LA can have an effect on the thermal stability. This issue could increase the strength of the material, justifying the effect on its degradation. However, this can affect its possible NPG linkage. The results raise doubts about the correct derivatization of NS-LA, since its maximum dTGA has not undergone great variation after 300 °C, and the modification could be minimal or simply some complexed LA residue may have remained.

To verify this hypothesis, an incubation of NS-LA with DTT was carried out, giving a soluble product (while NSs are insoluble, data not showed), destroying the polymer. Additionally, to confirm the presence of LA, an elemental analysis of RNS-LA was carried out, showing an S percentage, C = 40.21 ± 0.18, N = 2.03 ± 0.02, H = 5.69 ± 0.10, and S = 0.58 ± 0.01. In addition, the DLS of the three polymers was measured (Table S2 and Figure S1 in Supporting Information). The results showed that the three were similar in size to classical NS-CDI. An interesting point is the increase of the *Z*-average of the particle in NS-LA in comparison to the plain material, while for RNS-LA occurred the opposite. This fact can be attributed to the extra R–S–S–R′ bonding of NS-LA, which could increase the particle size, while a reducing agent may affect the typical nanosponge polymerization, generating a smaller material.^21,22^ For these reasons, the polymer RNS-LA was selected for the next steps of the research.

### 3.3 RNS-LA 1:8:0.1:0.1 Bonding to Nanoporous Gold

Once the polymer was prepared, the next step was to study the direct cleavage of RNS-LA to nanoporous gold. The first step was to confirm the capacity of LA to link NPG. The incubation of NPG in the presence of free LA at different temperatures was carried out, measuring the remaining LA in solution several times, selecting 55 °C as a gentle temperature for the experiment. Fragments of 60 mm^2^ were mixed with a suspension of RNS-LA at 3600 ppm for 72 h at 55 °C. The starting concentration of RNS-LA was fixed considering the calibration curve of RNS-LA and the sample dilution in that step. The results demonstrated that 24 h was enough to achieve the bonding equilibrium (Figure 4a). The RNS-LA_NPG was washed twice for 10 min to remove the adsorbed RNS-LA, which was subtracted to the quantity removed of the supernatant, giving a 60% of RNS-LA bonded to NPG by covalent link. The calculated mg/area was 1.1 × 10^–3^ mg/mm^2^.

**Figure 4:**
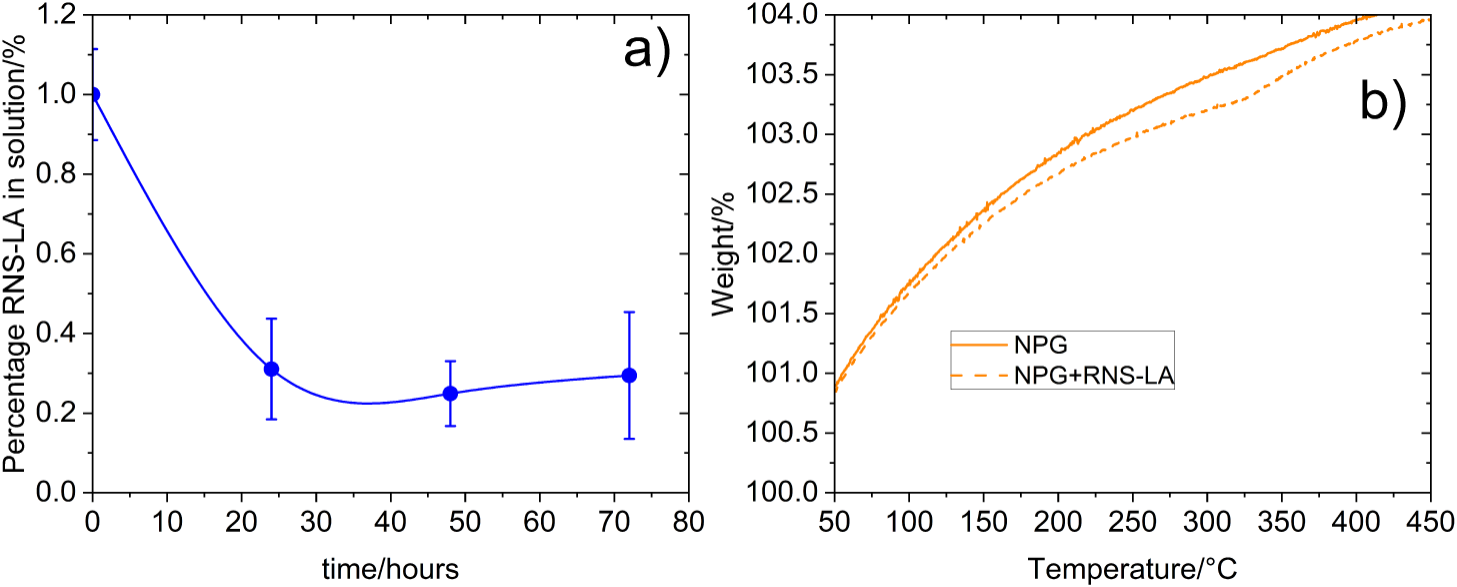
a) RNS-LA remaining under incubation with NPG. b) Thermogram of NPG with RNS-LA in comparison to free NPG.

To verify the correct bonding of the material, TGA and SERS analyses were carried out (Figures 4b and Figure 5). Although the TGA showed an overall increase in weight, possibly due to N_2_ adsorption,^23^ a slight weight loss was observed in RNS-LA_NPG around 320 °C (Figure 4b). This temperature is close to the dTGA maximum of RNS-LA, where the degradation rate reaches its maximum. This result supports the idea of a correct bonding of RNS-LA to NPG. Additionally, the SERS spectra of RNS-LA to NPG are reported in Figure 5.

**Figure 5:**
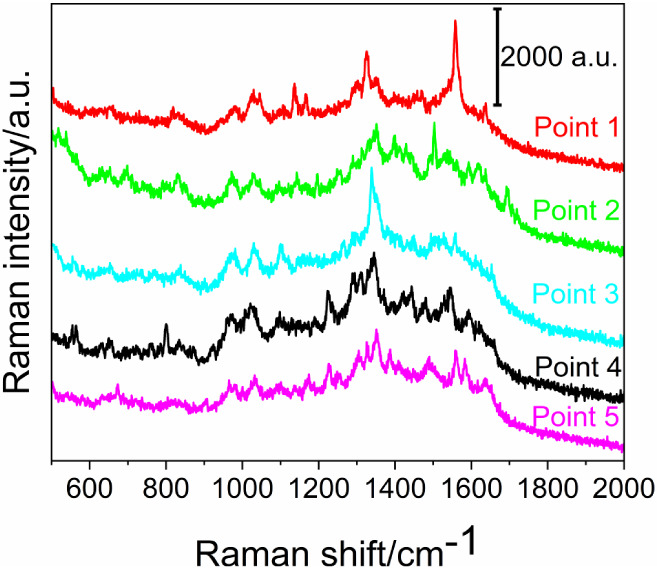
SERS spectra of RNS-LA to NPG, randomly collected on the sample surface.

The recorded spectra display numerous peaks, several of which remain challenging to assign with certainty. As reported in recent literature, β-cyclodextrins (β-CDs) do not exhibit significant Raman activity;^24,25^ consequently, the analysis was directed toward the identification of signals associated with lipoic acid and the carbonyl groups involved in cross-linking. Peak assignments were made based on previously published characterizations of lipoic acid adsorbed onto gold films:^26^ in the range 950–1100 cm^–1^ stretching C–C, 1100–1400 cm^–1^ deformation and torsion C–H, and at 1640 cm^–1^, stretching C=O.

### 3.4 Sensor Proof of Concept: Methylene Blue Complexes with RNS-LA_NPG

Once the RNS-LA_NPG had been correctly synthesized, the proof of concept with an organic pollutant was studied. Methylene blue (MB) was selected as an organic pollutant model due to its easy determination by UV and SERS.^27^

Although several studies have investigated methylene blue interactions with plain cyclodextrins,^28^ the interaction with RNS-LA has not been fully elucidated; therefore, the TGA and FTIR analyses of the complex were performed. The TGA in Figure 6a showed a shift in the dTGA maxima from 318 to 329 °C, due to the complexation of MB, indeed, the additional peaks of degradation of MB vanished, as usually occurs when a guest is complexed.^20,29^ In addition, the FTIR in Figure 6b showed few differences between RNS-LA and the MB-loaded RNS-LA, as the change in the peak in 1140 cm^–1^ could be due to N–CH_3_ stretching.^30^ All these points confirmed the capacity of RNS-LA to complex MB. Finally, in the conditions studied, the Removal efficiency and Adsorption capacity were 10.50 ± 0.05% and 1.50 ± 0.01% respectively.

**Figure 6:**
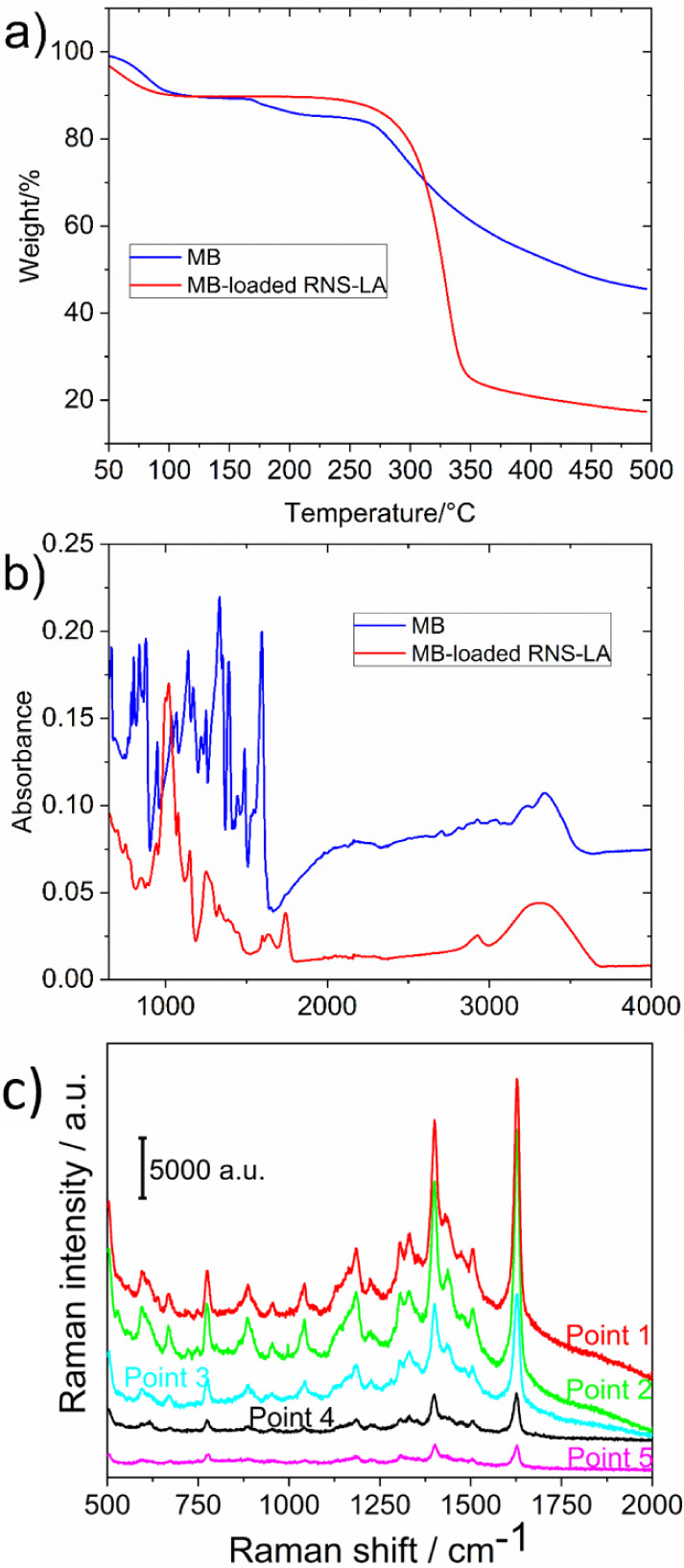
a) TGA of the MB (blue line) and MB-loaded RNS-LA (red line); b) FTIR of the MB (blue line) and MB-loaded RNS-LA (red line); c) SERS spectra of the methylene blue complexes with RNS-LA_NPG randomly collected on the sample surface.

After confirming the ability of the polymer to complex the model pollutant, the hybrid platform was evaluated in terms of its comparative retention capability. A methylene blue concentration of 3 ppm was selected, as it is representative of levels reported for some emerging contaminants such as caffeine.^31^ The functionalized and nonfunctionalized NPG substrates were incubated in a 3 ppm of MB solution in 0.8% NaCl for 30 min and 24 h. As shown in TableS3 in Supporting Information, the presence of RNS-LA on NPG results in a higher retention of MB compared to bare NPG, with an increase from 2.12% after 30 min up to 19.08% after 24 h. While the difference between both materials at shorter times is limited, it is statistically significant and becomes more pronounced at longer incubation times, reflecting the contribution of the nanosponge layer to enhanced analyte accumulation.

It should be noted that the observed increase in methylene blue retention (up to +19% after 24 h) reflects a comparative enhancement relative to bare NPG rather than an optimized sensing performance. In this context, the term “response” refers to the increased interaction of the contaminant with the functionalized surface, mediated by the nanosponge layer. The present system is conceived as a proof of concept operating at the interface between preconcentration and sensing, where diffusion and complexation processes dominate the kinetics. Although the response magnitude and time scale are not yet suitable for real-time applications, the results clearly demonstrate the functional contribution of the nanosponge to the hybrid platform.^7^ The difference is accentuated at long times, likely aided by the greater diffusion the molecule can achieve both in the NPG structure and in the structure of the polymer itself.^7^ The SERS analysis illustrated the union of MB into the composite (Figure 6c).

The signal at 775 cm^–1^ is attributed to the stretching vibrations of C–N bonding. The stretching mode is also responsible for the peak observed near 1400 cm^–1^. The peak at approximately 1050 cm^–1^ is likely associated with C–S stretching. In the spectral range between 800 cm^–1^ and 1400 cm^–1^, additional signals are present, corresponding to various vibrational modes involving C–H, C–C, and C–N bonds. The peak at 1620 cm^–1^ is assigned to the C=N stretching vibration of the heteroaromatic ring.^32,33^

These data support the use of RNS-LA to enhance the NPG response capacity to new contaminants.

The reproducibility of the SERS response was evaluated by acquiring two SERS maps on two independently prepared RNS-LA_NPG samples, fabricated under identical conditions and incubated overnight in a 10^–4^ M MB aqueous solution. The maps were generated based on the intensity of the most prominent Raman band at 1620 cm^–1^, assigned to the C=N stretching vibration of the heteroaromatic ring.^32,33^ Each map consisted of 36 measurement points, collected using the same acquisition parameters as for single-spot measurements, over a 100 × 100 μm^2^ area with a lateral step size of 20 μm.

The maps shown in Figure 7a and b highlight the presence of SERS hot spots randomly distributed over the substrate surface, reflecting local variations in the electromagnetic field enhancement. This heterogeneous spatial distribution is in agreement with the intrinsic morphology of the nanostructured substrate and it is consistent with variability reported in previous studies.^16,34^

**Figure 7:**
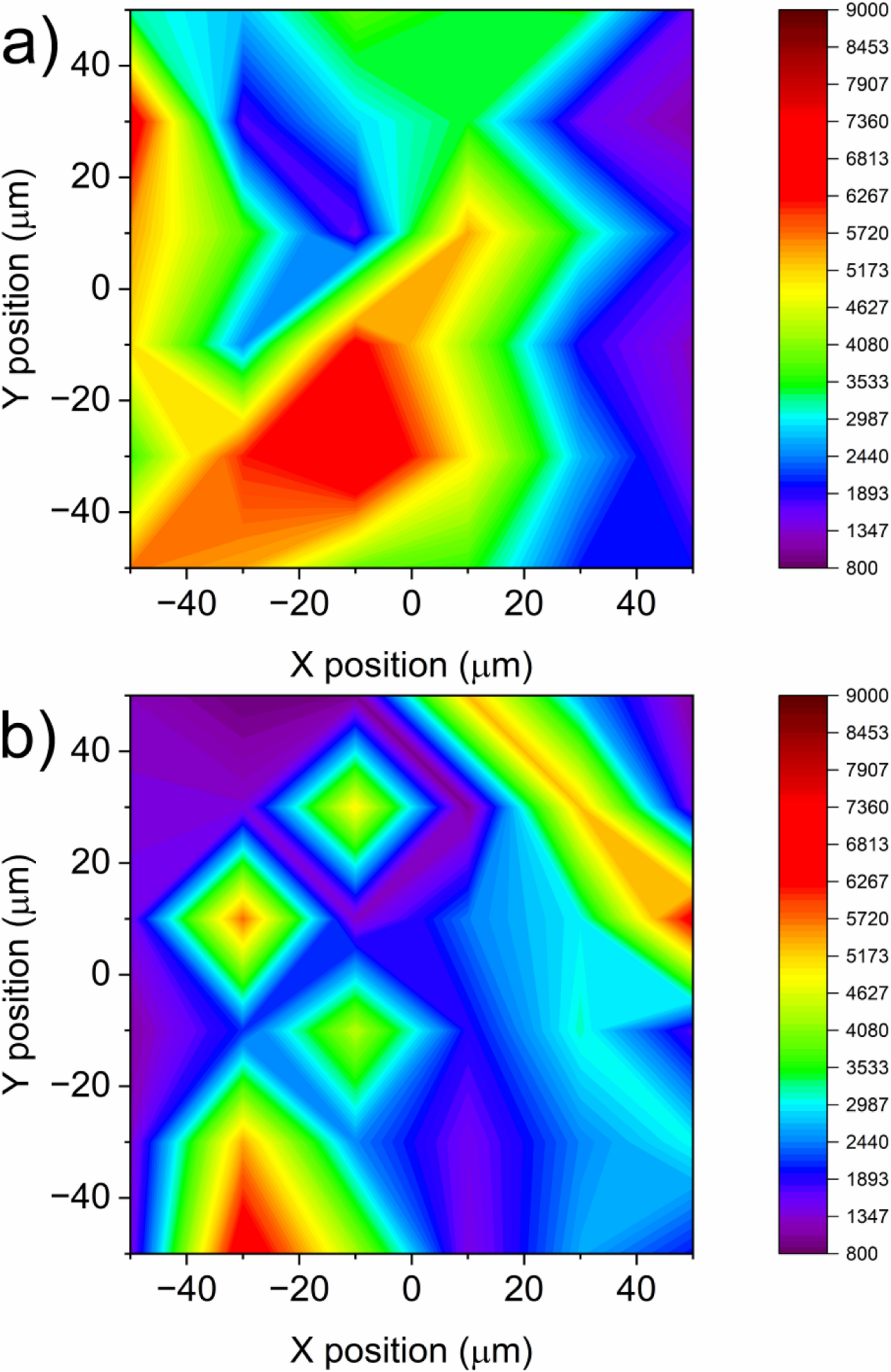
a) and b) represent SERS maps of two independently prepared RNS-LA_NPG substrates incubated overnight in a 10^–4^ M aqueous MB solution, constructed from the intensity of the 1620 cm^–1^ Raman band (C=N stretching).

As shown in Figure S2a and b in the Supporting Information, analysis of variance (ANOVA) demonstrates that the SERS responses obtained from the two independent trials, performed on different RNS-LA_NPG substrates prepared and measured under identical conditions, are statistically consistent, with no significant differences in substrate performance.

### 3.5 Practical Considerations and Limitations

From a practical perspective, several factors should be considered when evaluating the potential translation of the proposed system beyond the proof-of-concept stage. The nanoporous gold employed in this work, obtained by dealloying of an amorphous precursor, has been extensively investigated in previous studies and has demonstrated excellent SERS performance and well-defined structural features, making it a reliable and reproducible platform for functionalization.^35,36^ The precursor composition contains a limited fraction of noble metals (i.e., 20 at. % Au, 7 at. % Ag, and 5 at. % Pd), and the dealloyed ribbons have a thickness of about 20–25 μm, resulting in a low material usage per sample.

Based on current market prices of the constituent elements,^37,38^ gold is on the order of ∼150 USD g^–1^ (≈130 € g^–1^), palladium ∼50 USD g^–1^ (≈45 € g^–1^), silver ∼2.5 USD g^–1^ (≈2.2 € g^–1^), copper ∼0.01 USD g^–1^ (≈0.009 € g^–1^), and silicon contributing only marginally (∼1–2 USD g^–1^, ≈0.9–1.8 € g^–1^), the estimated cost of the Au_20_Cu_48_Ag_7_Pd_5_Si_20_ precursor alloy is on the order of ∼70 USD g^–1^ (≈65 € g^–1^). Considering the small amount of material employed per sample unit, this corresponds to approximately ∼0.7 USD (≈0.6–0.7 €) for 10 mg of alloy. Therefore, although the use of gold remains a relevant cost factor for large-scale applications, the effective material cost per sample is significantly mitigated by both the limited noble metal content and the small mass involved.

Nanoporous gold was selected for its superior chemical stability and reproducibility, particularly under aqueous conditions, where silver-based substrates are more prone to degradation. This choice is consistent with the aim of establishing a robust proof-of-concept system, although alternative materials may be considered in future developments to improve the cost efficiency.

The dealloying process involves nitric acid and a limited concentration of hydrofluoric acid (0.5 M), with conditions optimized to achieve the desired morphology. While such treatments require careful handling and may limit straightforward scalability, the volumes used at the laboratory scale are relatively small. Similarly, piranha cleaning is performed using limited volumes sufficient for multiple samples. In contrast, the synthesis of CD-NS is carried out under aqueous conditions and does not pose significant environmental or safety concerns.

Overall, the present system demonstrates the feasibility of integrating nanoporous gold with functional polymeric components, while further optimization is required for scalable and application-oriented implementations. In particular, the relatively long interaction times observed in the current study (up to 24 h) highlight the need for further improvements to enable faster and more practical sensing and treatment processes. It should also be noted that the concentrations investigated in this study fall within the parts per million range and are therefore not representative of many trace levels typically encountered in environmental monitoring. In this respect, the present results are not intended to demonstrate trace-level detection capabilities. Moreover, the experiments were carried out in model aqueous systems, and matrix effects in more complex environments (e.g., tap water, river water, or wastewater) were not evaluated. These aspects represent the limitations of the current study and will require further investigation.

## 4 Conclusions

In conclusion, this work presents an interesting proof-of-concept study on the first-ever binding of nanosponge polymer to nanoporous gold. Specifically, the polymer was derivatized with a biodegradable lipoic acid linker to efficiently bind to gold via sulfide bonds. Two synthesis attempts were made, but the presence of reducing agents was necessary during nanosponge preparation to obtain reduced −SH groups that could subsequently bind to NPG. The polymer was able to bind with an mg/area ratio of 1.1 × 10^–3^ mg/mm^2^, confirmed by both SERS and colorimetric methods. Using methylene blue as a model molecule, a proof-of-concept study of its detection was performed, obtaining a 19% higher response than gold alone. These data lay the groundwork for the union of both materials for the detection and possible elimination of emerging contaminants in water.

## Supplementary Material



## Data Availability

Data will be made available on request.

## #

Anton Paar Italia S.r.l., Via Albenga 78, 10098 Rivoli, Italia
