# Supplementary material for: Hybrid Nanoporous Gold–Cyclodextrin Nanosponge Platforms for Enhanced Model Contaminant Detection
Source: ACS Appl Polym Mater. 2026 May 11;8(10):7682–90. doi: 10.1021/acsapm.6c00986 (PMC13206502; doi:10.1021/acsapm.6c00986)
Supplement: Supplementary file 1 [file ap6c00986_si_001.pdf]

Supporting Information

HYBRID NANOPOROUS GOLD–CYCLODEXTRIN NANOSPONGE PLATFORMS FOR ENHANCED MODEL CONTAMINANT DETECTION

Adrián Matencio [1,2]; Federico Scaglione [1*]; Miriam Birolo [1]; Alberto Rubin-Pedrazzo [1,ε];

Paola Rizzi [1]; Francesco Trotta [1]; Fabrizio Caldera [1*]

1 Department of Chemistry and NIS - INSTM, University of Turin, Via P. Giuria 7, 10125 Turin, Italy.

2 Departamento de Bioquímica y Biología Molecular-A, Facultad de Biología, Universidad de Murcia–Regional Campus of International Excellence“Campus Mare Nostrum”, E-30100, Murcia, Spain

ε Current affiliation: Anton Paar Italia S.r.l. Via Albenga 78, 10098 Rivoli, Italia

* Correspondence: author: (F. Scaglione: federico.scaglione@unito.it / F. Caldera: fabrizio.caldera@unito.it)

Table S1. EDS analysis of NPG.

at. %	Au	Cu	Ag	Pd	Si
Mean ± SD	93.3±0.2	5.3±0.2	0.1±0.1	0.3±0.2	1.0±0.1

Table S2. Values of Z-average and PDI (polydispersity) of the samples, with their SMD (±). Due to the high polydispersity, the Z-average value should be considered only indicative.

Sample	*Z-Average (nm)*	*SMD*	*PDI*	*SMD*
βNS-CDI 1:8	564.00	13.00	0.34	0.03
NS-LA 1:8:0.1	689.00	35.00	0.57	0.01
RNS-LA 1:8:0.1:0.1	380.00	62.00	0.27	0.12

Figure S1

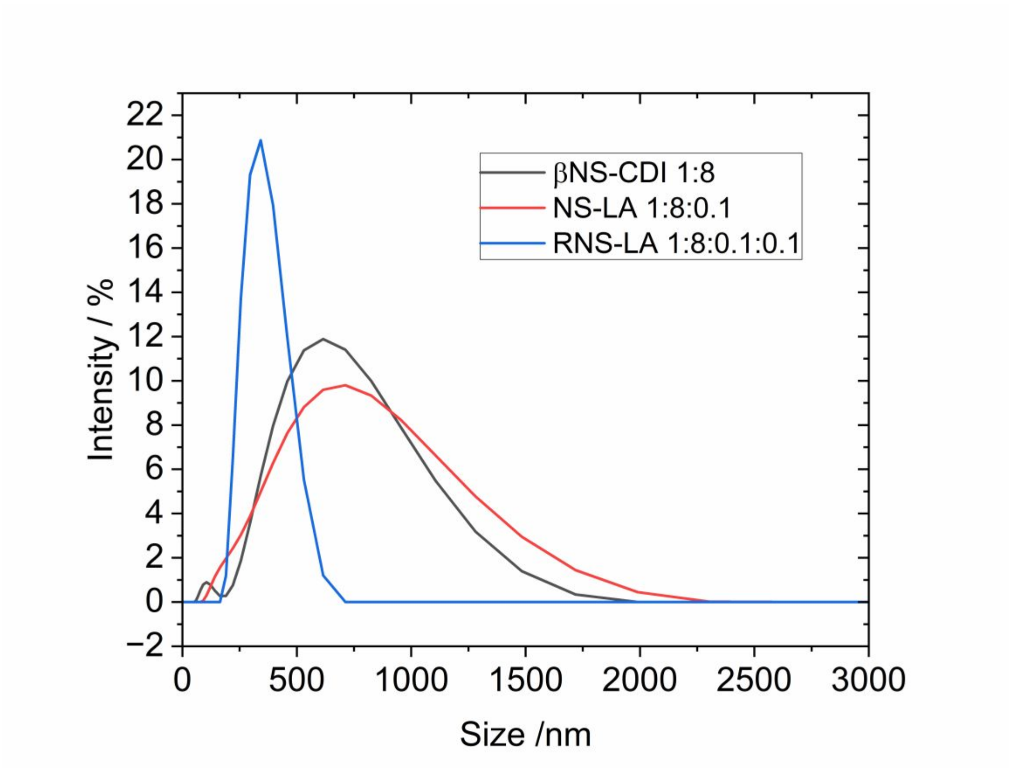

Figure S1. Representative DLS size distribution of βNS-CDI 1:8, NS-LA 1:8:0.1 and RNS-LA 1:8:0.1:0.1 until 3000 nm. Three independent measurements were performed, and the reported

Z-average hydrodynamic diameter and PDI values correspond to the mean ± SMD (see Table S2).

Due to the high polydispersity of the system, the distribution is shown as representative.

Table S3. Comparative retention of Methylene Blue (MB, normalized with the initial quantity) by NPG and RNS-LA_NPG

Sample	***0.5h***	***SD***	***24h***	***SD***
MB + NPG	*4.59%*	*0.23%*	*25.09%*	*1.58%*
MB + RNS-LA_NPG	*6.71%*	*0.34%*	*44.17%*	*2.78%*

Figure S2

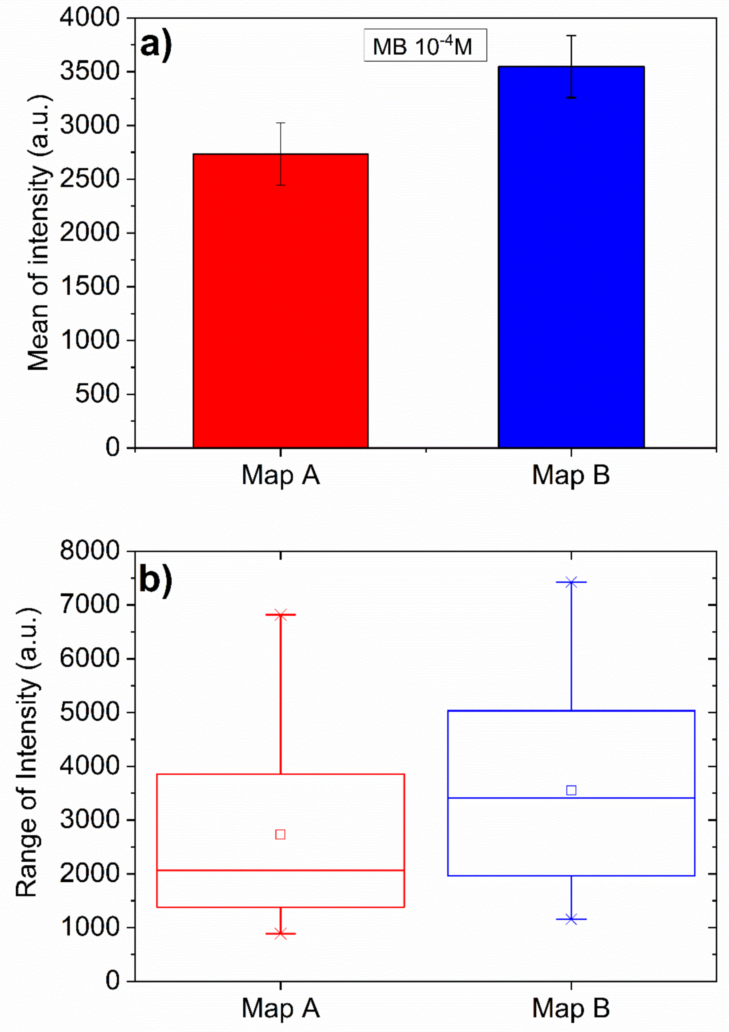

Figure S2. a) Mean SERS intensity ± standard error (SE) obtained from the two maps at an MB concentration of 10^{-4} M; (b) ANOVA (analysis of variance) plot comparing the intensity distributions of the two maps.
